# Composites Based on Poly(ε-caprolactone) and Graphene Oxide Modified with Oligo/Poly(Glutamic Acid) as Biomaterials with Osteoconductive Properties

**DOI:** 10.3390/polym15122714

**Published:** 2023-06-17

**Authors:** Olga Solomakha, Mariia Stepanova, Iosif Gofman, Yulia Nashchekina, Maxim Rabchinskii, Alexey Nashchekin, Antonina Lavrentieva, Evgenia Korzhikova-Vlakh

**Affiliations:** 1 Institute of Macromolecular Compounds, Russian Academy of Sciences, St. Petersburg 199004, Russia; solomanya@bk.ru (O.S.); maristepanova@hq.macro.ru (M.S.); gofman@imc.macro.ru (I.G.); 2 Institute of Cytology, Russian Academy of Sciences, St. Petersburg 194064, Russia; ulychka@mail.ru; 3 Ioffe Institute, Politekhnicheskaya St. 26, St. Petersburg 194021, Russia; rabchinskii@mail.ioffe.ru (M.R.); nashchekin@mail.ioffe.ru (A.N.); 4 Institute of Technical Chemistry, Leibniz University of Hannover, 30167 Hannover, Germany; lavrentieva@iftc.uni-hannover.de

**Keywords:** graphene oxide, graphene oxide modification, grafting from, grafting to, oligomers and polymers of glutamic acid, poly(ε-caprolactone), biocompatible polymer composites, osteoconductive materials

## Abstract

The development of new biodegradable biomaterials with osteoconductive properties for bone tissue regeneration is one of the urgent tasks of modern medicine. In this study, we proposed the pathway for graphene oxide (GO) modification with oligo/poly(glutamic acid) (oligo/poly(Glu)) possessing osteoconductive properties. The modification was confirmed by a number of methods such as Fourier-transform infrared spectroscopy, quantitative amino acid HPLC analysis, thermogravimetric analysis, scanning electron microscopy, and dynamic and electrophoretic light scattering. Modified GO was used as a filler for poly(ε-caprolactone) (PCL) in the fabrication of composite films. The mechanical properties of the biocomposites were compared with those obtained for the PCL/GO composites. An 18–27% increase in elastic modulus was found for all composites containing modified GO. No significant cytotoxicity of the GO and its derivatives in human osteosarcoma cells (MG-63) was revealed. Moreover, the developed composites stimulated the proliferation of human mesenchymal stem cells (hMSCs) adhered to the surface of the films in comparison with unfilled PCL material. The osteoconductive properties of the PCL-based composites filled with GO modified with oligo/poly(Glu) were confirmed via alkaline phosphatase assay as well as calcein and alizarin red S staining after osteogenic differentiation of hMSC in vitro.

## 1. Introduction

Reconstruction of bone tissue and optimization of the regeneration process is one of the urgent tasks in orthopedics, traumatology, and bone surgery related to bone diseases (e.g., osteosarcoma or bone tuberculosis) that require removal of part of the bone tissue with subsequent repair of a defect [1]. Recent strategies in this area focus on the development of biodegradable/bioresorbable and biocompatible materials [2] such as bioceramics [3] and natural [4,5,6,7] and synthetic polymers [8,9].

Bioceramics are the oldest type of materials proposed for bone regeneration [3,10,11]. They demonstrate excellent biocompatibility and good osteoconductivity accompanied by physicochemical properties close to bone mineral components. At the same time, their drawbacks include slow resorbability, brittleness, and mediocre mechanical properties. Materials based on natural polymers are represented by proteins (collagen, gelatin, silk, etc.), polysaccharides (chitosan, alginate, hyaluronic acid, etc.), and polyhydroxyalkanoates (poly(3/4-hydroxybutirate), poly(3-hydroxybutirate-co-3-hydroxyvalerate), etc.). Protein-based materials demonstrate higher cell adhesion and osteoconductivity compared to polysaccharide and polyhydroxyalkanoate ones [12]. However, these polymers have non-reproducible properties from batch to batch and can contain the impurities of endotoxins. Both protein and polysaccharide materials exhibit poor mechanical properties, while polyhydroxyalkanoates exhibit low cell adhesion [13,14] and no osteoconductivity [15,16]. At the same time, the mechanical properties of polyhydroxyalkanoates are better than those of other natural polymers.

Synthetic polymers belonging to the class of aliphatic polyesters (or synthetic polyhydroxyalkanoates) are biodegradable and biocompatible, and they are produced by ring-opening polymerization of cyclic monomers. Polymers such as poly(L/D,L-lactic acid) (PLA), poly(lactic acid-co-glycolic acid), and poly(ε-caprolactone) (PCL) are among the most studied aliphatic polyesters that are approved for biomedical applications [17,18,19]. Despite their ability to degrade in the biological environment to non-toxic products and materials, these polymers are rather hydrophobic, exhibit poor cell adhesion and mediocre mechanical properties, and have no osteoconductive properties [20,21]. For advanced medical applications, including bone regeneration, polyesters are modified in a variety of ways, including surface modification to increase cell adhesion and/or osteoinductivity [22,23], the incorporation of fillers into the polymer matrix to improve mechanical and osteoconductive properties [24,25,26], and the introduction of various drugs to impart bioactive properties of interest [27,28,29].

In the last decade, the positive properties and applicability of graphene and its derivatives for many applications in biomedicine have been discovered [30,31,32]. In particular, the application of graphene [33,34] and its derivatives [35,36,37] as filler to PLA has been shown to improve the mechanical properties of PLA-based materials. Moreover, the introduction of hydrophilized graphene, such as graphene oxide (GO) or reduced graphene oxide (rGO), enhances the positive interaction of cells with GO-containing composites that favors cell growth, proliferation, and differentiation [32,38] as well as provides osteoconductive properties [39].

One of the main obstacles in the preparation of composites is the aggregation of the filler in the polymer matrix [40,41]. For graphene and its derivatives, the pronounced aggregation and, as a consequence, poor distribution in the polymer matrix can be the result of strong π–π interaction. However, GO and rGO demonstrate enhanced stability in water and polar solvents compared to non-modified graphene. Surface modification is one of the known approaches to regulate the properties of the filler for its better distribution in the polymer matrix [42,43,44]. Recently, Gu et al. reported the functionalization of GO via polyamide in ethanol and further application for preparing nanocomposites using melt spinning [45]. Such nanocomposites demonstrated excellent mechanical properties. Furthermore, it was shown that modifying GO with PLA also contributed positively to improved compatibility of the filler with the PLA matrix [25].

In our recent papers, we have shown the positive effect of modifying nanocrystalline cellulose with poly(α,L-glutamic acid) (poly(Glu)) to enhance the filler compatibility with PLA and PCL [46,47] and provide osteoconductive properties to the composite materials due to the presence of poly(Glu) [48]. In addition, the enhancement in mechanical properties was also shown for aminated graphene modified with oligomers of L-glutamic acid (oligo(Glu)) [35].

In this study, various ways of modifying GO with oligo/poly(Glu) were proposed. In particular, the “grafting from” and “grafting to” approaches were implemented for GO modification with oligo/poly(Glu). Modification of GO with oligo/polyGlu can allow a better distribution of GO in the matrix of the hydrophobic polymer and give it more pronounced osteoconductive properties. “Grafting from” was performed via ring-opening polymerization of N-carboxyanhydride of γ-benzyl ester of glutamic acid initiated by primary amino groups introduced into GO via its pre-modification with ethylene diamine or L-lysine as linkers. “Grafting to” was carried out with the use of pre-synthesized and activated poly(Glu) via its reaction with primary amino groups introduced into GO as in the previous case. The resulting modified samples were carefully characterized using a number of methods to verify poly(Glu) grafting and to evaluate changes in morphology, size, and properties of GO as a filler for PCL. Composites containing unmodified GO were involved in the study as control materials to investigate the effects of modification.

The in vitro biocompatibility of both dispersed GO samples and PCL-based composites containing unmodified and functionalized GO as fillers were evaluated in MG-63 cells (human osteosarcoma cells). The proliferation of human mesenchymal stem cells (hMSCs) on the surface of the polymer films as well as osteodifferentiation and biomineralization were examined to evaluate the osteoconductive properties of the developed composites.

## 2. Materials and Methods

### 2.1. Chemicals, Supplements, and Cells

The aqueous dispersion of GO (0.6 wt%) was a product of Graphene Technologies (Moscow, Russia, www.graphtechrus.com, accessed 11 May 2023). The content of epoxy groups in the dry phase of GO was 8 mmol/g (according to X-ray photoelectron spectroscopy and calculations similar to those described elsewhere [36]). *γ*-Benzyl-L-glutamate (Glu(OBzl)) (>99%), *n*-hexylamine (98%), α-pinene (98%), triphosgene (98%), *N^ε^*-(tert-butoxycarbonyl)-*L*-lysine (Lys(Boc), ≥95%), 1-hydroxybenzotriazole hydrate (HOBT, ≥97%), *N*,*N′*-diisopropylcarbodiimide (DIC, 99%), calcein–AM (≥98%), and alizarin red S were purchased from Sigma-Aldrich (Darmstadt, Germany). *N*-Boc-ethylenediamine (EDA(Boc)), 98%), 5-bromo-4-chloro-indolyl phosphate tetrazole blue (BCIP–NBT) was supplied by Ark Pharm Inc. (Arlington Heights, IL, USA). Calcein (≥98%) was a product of Lenreaktiv Ltd. (St. Petersburg, Russia). Salts for buffer solution preparation, hydrochloric acid (concentrated), sodium hydroxide, and phenol (all ≥99% purity) as well as organic solvents used in this work were products of Vecton Ltd. (St. Petersburg, Russia). All organic solvents were distilled before application. In addition, dimethyl sulfoxide (DMSO) and methanol used for GO modification and Boc deprotection, respectively, were preliminarily dried using 4 Å molecular sieves.

Nylon and hydrophobic PTFE syringe filters with a pore size of 0.22 and 0.45 µm, respectively, and a diameter of 13 mm were supplied by Nantong FilterBio Membrane (Nantong, China). BF-6 medical glue was a product of the Tula Pharmaceutical Company (Tula, Russia).

Low molecular weight poly(L-glutamic acid) (poly(Glu)) used to modify GO was synthesized via ring-opening polymerization of *γ*-benzyl-L-glutamic acid *N*-carboxyanhydride (Glu(OBzl) NCA) monomer and subsequent removal of benzyl protecting groups as described elsewhere [44]. *n*-Hexylamine was used as an initiator and the monomer/initiator molar ratio was 100. The resulting poly(Glu) had the following characteristics (according to ^1^H NMR spectroscopy): degree of polymerization (DP) = 72; M*_n_* = 11,100; and the content of residual benzyl groups was equal to 27 mol%.

Human osteosarcoma cells (MG-63) and fetal hMSCs used for cytotoxicity and proliferation study, respectively, were received from the Vertebrate Cell Culture Collection of Institute of Cytology RAS (St. Petersburg, Russia) and cultivated as described elsewhere [48,49]. Adipose hMSCs were received from the Cell Culture Collection of Institute of Technical Chemistry of Leibniz University of Hannover (Hannover, Germany) and cultivated in alpha-MEM medium (Thermo Fisher Scientific, Waltham, MA, USA) containing 10% CC-pro serum (Oberdorla, Germany) and 0.5% gentamicin (Merck Millipore, Darmstadt, Germany).

### 2.2. Synthesis of GO-graft-oligo(Glu) or GO-graft-poly(Glu)

Initially, GO was modified with L-lysine (Lys) or ethylenediamine (EDA) as described below. A solution of Lys(Boc) (175 mL, 6.5 mg/mL) dissolved in 0.01 M borate buffer (BB, pH 9.0) was added to the initial aqueous suspension of GO (400 mg), based on the molar ratio of GO epoxy groups to Lys(Boc) α-amino groups equal to 1/1.4. The reaction was carried out in an orbital thermoshaker Unimax 1010 (Heidolph, Schwabach, Germany) at 30 °C and 150 rpm for 24 h. The resulting modified GO particles (GO-Lys(Boc)) were separated and washed with water (20–25 mL each) using centrifugation (Sigma 2-16KL centrifuge, Sigma, Osterode am Harz, Germany) at 9 °C and 12,000 rpm for 30 min. Washing was carried out 10 times, after which the sediment was freeze-dried. Modification in the case of (Boc) was implemented in a similar manner, except that EDA(Boc) was mixed with 1,4-dioxane/BB (1/15, *v*/*v*) before adding this solution (260 mL, 2.8 mg/mL) to GO. The resulting product (GO-EDA(Boc)) was washed first with DMSO (4 times) and then with water (8 times), and finally freeze-dried. The weights of the dry products were 381 mg (~61% of the theoretical yield) and 387 mg (~71% of the theoretical yield) for GO-Lys(Boc) and GO-EDA(Boc), respectively.

To remove the Boc protecting groups, 11 mL of 4 M hydrochloric acid in dry methanol was added to GO-Lys(Boc)/GO-EDA(Boc) (370 mg). The reaction was carried out in a closed flask for 5 h at room temperature with constant stirring of the suspension. GO-Lys and GO-EDA obtained after deblocking were separated using centrifugation (9 °C, 12,000 rpm, 30 min), then washed sequentially with methanol (2 times) and water (10 times), frozen, and freeze-dried. The yield for GO-EDA/GO-Lys particles was ~97/98%. Next, the obtained particles of both types were modified with oligo/poly(Glu) using the “grafting from” or “grafting to” techniques.

“Grafting from” the surface of the obtained GO particles of oligomeric chains of glutamic acid was similar to the technique reported for the aminated graphene [32]. For this purpose, GO-Lys/GO-EDA modifications with oligo(Glu) were performed via ring-opening polymerization of the Glu(OBzl) NCA monomer on the surface of the modified GO, whose amino groups of Lys or EDA initiated polymerization. An amount of 50 mg of GO-Lys or GO-EDA were placed in a flask and dispersed in 2 mL of freshly distilled ethyl acetate for about 10 min at 18 kHz using an ultrasonic bath UZV1-0.16/18 (UST Ltd., St. Petersburg, Russia). After that, the system was purged with argon for 5 min. Next, a solution of an appropriate amount of Glu(OBzl) NCA (preliminarily purged with argon for 10 min) was added to the resulting suspension, and the flask with the reaction system was placed in an orbital thermoshaker (125 rpm). The reaction was carried out for 48–72 h at 35–45 °C and molar ratios of epoxy groups GO/monomer equal 1.6, 4.7, and 10. Using different excesses of the monomer, different volumes of ethyl acetate were added to the weighted components. Specifically, 3, 6, and 12 mL of ethyl acetate were added to 150, 450, and 900 mg of monomer for 1.6-, 4.7-, and 10-fold monomer excesses, respectively. At the end of the synthesis, the product was separated and washed with *N*,*N*’-dimethylformamide 4 times and with water 8 times using centrifugation followed by freeze-drying. The yields for GO-Lys-oligo(Glu(OBzl)) at different times and temperatures of synthesis in the case of 1.6, 4.7, and 10-fold excess of the monomer were 59–65%, ~81%, and 91–96%, respectively. In the case of GO-EDA-oligo(Glu(OBzl)) the yield was 45–67% (1.6-fold monomer excess). After that, the removal of benzyl protective groups was carried out to obtain GO-Lys-oligo(Glu)/GO-EDA-oligo(Glu) particles. For deprotection, the protocol described elsewhere was applied. After washing using centrifugation (4 times with DMSO and 8 times with water, 15 °C, 12,000 rpm, 30 min) and freeze-drying, the yields of GO-Lys-oligo(Glu) were 63–71% (1.6-fold monomer excess), ~60% (4.7-fold monomer excess) and 51% (10-fold excess of monomer). The yield of GO-EDA-oligo(Glu) was 45–58% (1.6-fold excess of monomer).

“Grafting to” GO surface was performed using the method of activated esters by attaching the pre-synthesized and deprotected poly(Glu). For this modification, the number of activated carboxyl groups of poly(Glu) was varied by 30 and 100%. Poly(Glu) (25 mg) was dissolved in 12 mL of dry DMSO, and then solutions of HOBT (0.5 mL, 91 mg/mL) and DIC (0.5 mL, 41 mg/mL) were added to the obtained solution in dry DMSO using a step-wise mode. The following molar ratio between reagents was used for modification: carboxyl groups of poly(Glu)/HOBT/DIC = 1/2/1. The reaction was carried out for 20 min under constant stirring with a magnetic stirrer MR Hei-Standard (Heidolph, Schwabach, Germany) at room temperature. The resulting system was transferred to a suspension (50 mg) of GO-Lys or GO-EDA dispersed in 3 mL of dry DMSO, and the modification reaction was carried out for 3 h under constant stirring at room temperature. The resulting GO-Lys-poly(Glu)/GO-EDA-poly(Glu) was precipitated using centrifugation (15 °C, 12,000 rpm, 30 min); then the product was successively washed and separated via centrifugation 5 times with DMSO (15 °C, 12,000 rpm, 30 min) and 10 times with water (10 °C, 12,000 rpm, 30 min) and freeze-dried. The yields for GO-Lys-poly(Glu) were 78 wt% for the 100% -COOH activation and 83 wt% for the 30% -COOH activation. The yield for GO-EDA-poly(Glu) modified under 100% activation of carboxylic groups was 69 wt%.

### 2.3. Characterization of GO Derivatives

Fourier-transform infrared spectroscopy (FTIR) was performed with the use of IRAffinity-1 S (Shimadzu, Kyoto, Japan). The FTIR spectra were recorded in the range of 500–4000 cm^−1^ with 40 scans at a resolution of 2 cm^−1^ using the KBr tablet technique (0.2 mg sample per 20 mg KBr).

Thermogravimetric analysis (TGA) was carried out with the use of a DTG-60 instrument (Shimadzu, Kyoto, Japan) at a heating rate of 5 °C/min in the range from 40 to 700 °C (air atmosphere).

Hydrodynamic diameter (*D_H_*) and electrokinetic (zeta) potential of GO particles before and after their modification were estimated using dynamic and electrophoretic light scattering using a ZetasizerNano-ZS (Malvern Panalytical, Malvern, UK) with an installed He–Ne laser (633 nm). The measurement conditions were as follows: scattering angle 173°, sample concentration 0.5 mg/mL, water alkalized with 0.1 M NaOH solution to pH 7.5 for *D_H_* determination and water for zeta-potential measurements, 25 °C. Before the study, all samples were dispersed with the ultrasonic probe Sonopuls MS 73 (Bandelin, Berlin, Germany) for 10 s at a power of 40%.

The contents of Lys and Glu in the modified GO were determined using quantitative HPLC analysis of free amino acids produced after total acidic hydrolysis of the modified GO samples. Hydrolysis and HPLC analysis were performed as described in our previous work, with the only difference in the sample weight used for hydrolysis [50]. In the case of modified GO, the sample weight used for hydrolysis was 6–9 mg.

### 2.4. Synthesis of PCL

Poly(*ε*-caprolactone) (PCL) was prepared using *in bulk* ring-opening polymerization of *ε*-caprolactone using methanol as initiator and tin(II) octoate as catalyst according to the protocol published elsewhere [49]. The synthesis was carried out in an argon atmosphere at 130 °C for 24 h with a monomer/methanol/tin(II) octoate molar ratio of 4900/2/1. Molecular weight characteristics (M*_n_* and *Đ*) and intrinsic viscosity (*η*) of the resulting PCL were determined exactly as described in our previous research [49].

### 2.5. Manufacturing of Composite Films

The composite and unfilled (as control) PCL-based films were produced according to the previously developed procedure [48]. Briefly, a PCL chloroform solution (5%, 6.8 mL) filtered through a PTFE syringe filter was cast onto cellophane film stretched over a glass ring (76 mm i.d., 20 mm height) and dried in the air at room temperature to remove the solvent (12–14 h). Then, the polymer film was removed from the supporting cellophane, placed in a Petri dish, and additionally dried in the air with the thermostat at 40 °C until constant weight (about 7 days). In the case of composite films, 0.5 and 1 wt% (relative to the polymer) of unmodified or modified GO particles were dispersed in a 5% PCL solution in CHCl_3_ in an ultrasonic bath with ice for 10–15 min. After that, solution casting followed by solvent evaporation were performed as described above for unfilled PCL. The scheme of film manufacturing is illustrated in Figure S1 (Supplementary Materials).

### 2.6. Microscopic Analysis

The morphology of GO particles before and after modification, as well as the prepared polymer films, was examined using scanning electron microscopy (SEM) using a JSM-7001F Jeol (Tokyo, Japan) microscope. The GO samples for SEM analysis were prepared by placing 0.005 wt% of a GO ethanol dispersion onto a silicon substrate and further drying them. In the case of film specimens, conductive gold layers were preliminarily deposited on their surfaces.

Fluorescence microscopy of samples after mineralization in a model media and cell proliferation as well as osteogenic cell differentiation were performed using a Mikmed-2 microscope (Lomo, St.-Petersburg, Russia) and a BioTek Cytation 5 Cell Imaging Multimode Reader (Agilent, Santa Clara, CA, USA), respectively.

Optical microscopy was carried out using the Nikon Eclipse E200 microscope (Tokyo, Japan) in transmission mode.

### 2.7. Mechanical Properties

Mechanical testing of unfiled PCL and PCL-based composite films was performed according to ASTM D638 requirements in the uniaxial tension mode at an extension rate of 10 mm/min using an AG-100X Plus universal mechanical tests system (Shimadzu, Kyoto, Japan). For examination, film strips with a length of 20 mm and width of 2 mm and a thickness of 92 ± 11 μm were used. The characteristics of the films were evaluated and averaged from the results of testing for 7–9 strips of each kind of material. The study was carried out at room temperature (20 °C). The maximal load values applied to the film samples during the tests (the load at the yield point) were from 2 to 3 N. The test system was equipped for these tests with the Shimadzu 339-83459-07 load cell kit (nominal capacity 50 N, class 0.5).

### 2.8. Cytotoxicity, Proliferation, and Biomineralization

#### 2.8.1. Cytotoxicity

The cytotoxicity of modified GO particles was tested in MG-63 cells for 72 h in the range of concentrations from 4 to 1000 µg/mL using an MTT assay as described in our previous paper [35].

In order to compare the adhesion and proliferation of cells on the surface of composite films, the UV-sterilized film specimens with a diameter of 6 mm (*n* = 3) were glued to the bottom of the wells of a 96-well plate using BF-6 medical glue (Tula pharmaceutical factory, Tula, Russia). Then, 5 × 10^3^ fetal hMSCs were seeded in each well. Cells were cultured at 37 °C in a humidified atmosphere containing 5% CO_2_ in DMEM-F12 medium (Dulbecco’s modified Eagle’s medium; Gibco, Life Technologies, Paisley, UK) supplied with 1% essential amino acids, 10% (*v*/*v*) thermally inactivated fetal bovine serum (FBS; HyClone, Logan, UT, USA), 1% L-glutamine, 50 U/mL penicillin, and 50 μg/mL streptomycin. The cultivation was performed within 3 and 7 days. After the predetermined time, MTT (Sigma, St. Louis, MO, USA) solution was added to each well and incubated for 2 h at 37 °C in the atmosphere with 5% CO_2_. After the incubation time, the above solution was removed, and the colored formazan crystals were solubilized by adding 50 μL of dimethyl sulfoxide. The absorbance was measured at 570 nm in a multiwell plate reader (Thermo Fisher Multiscan Labsystems, Waltham, MA, USA). The absorbance data were processed using Microsoft Excel 2019 software (ver. 16.0.12527.22261, Redmond, Washington, DC, USA).

#### 2.8.2. ALP Assay

The UV-sterilized film specimens (*n* = 3) with a diameter of 10 mm were glued to the coverslip glasses of the same diameter using BF-6 medical glue and placed into the wells of a 24-well plate. Then, 4 × 10^4^ fetal hMSCs were seeded in each well. Cells were cultivated as described in Section 2.8.1. Proliferation was performed for 3–4 days until the confluence of 90% was reached. After that, the medium was changed to differentiation MSCgo™ 05-440-1B medium (Biological Industries, Beit-Haemek, Israel) containing 10% (*v*/*v*) FBS; 1% L-glutamine; 50 U/mL penicillin; and 50 μg/mL streptomycin, β-glycerophosphate, dexamethasone (1000×), and ascorbic acid. After 14 days of cultivation, cells were washed three times with PBS and fixed with 4% formaldehyde solution for 30 min at room temperature. The cells were washed three times with PBS for 5 min each and stained for alkaline phosphatase with BCIP–NBT mixture for 30–60 min in the dark at room temperature. The stained preparations were washed several times with distilled water and dried.

#### 2.8.3. Biomineralization Study

The UV-sterilized polymer films of 6 mm in diameter were glued to the bottom of the wells (3 pcs/well) in a 6-well plate using BF-6 medical glue. Then, 8 × 10^4^ adipose hMSC cells were added to each well and cultured for 10 days in an alfa-MEM supplied with 10% of serum and in a humidified atmosphere (5% CO_2_, 37 °C). The adhesion and proliferation of cells on the surface were confirmed by staining the cells on the surface of the films with the calcein–AM. For this, the medium was removed from the wells, and 1 mL of a preincubated calcein–AM solution (4 µg/mL) in LHC Basal Medium (1×) (Gibco, Life Technologies, Paisley, UK) was added to each well and incubated for 10 min for cell staining. After that, all specimens were examined using fluorescence microscopy (Section 2.6).

After 10 days of cultivation, the culture medium was changed to osteogenic differentiation medium (ODM) supplemented with 2.5% human platelet lysate, 5 mM beta-glycerol phosphate, and 0.2 mM L-ascorbate-2-phosphate, in which cells were cultured for 19 days. After that, the cells were fixed in a 4% aqueous solution of paraformaldehyde and stained with a fluorescent dye calcein or alizarin red S. Each film composition was analyzed in a series of three specimens.

*Calcein staining*: 1 mL of calcein solution in water with concentration of a 5 µg/mL was added to the well of the plate containing the glued film specimens. Then, specimens were incubated overnight in the dark at 4 °C and finally washed thoroughly with water (approximately 5 times with 4 mL). Calcein fluorescence was detected at the emission wavelength 530 nm (an excitation wavelength is 480 nm) using fluorescence microscope (Section 2.6).

*Alizarin red S staining*: 1 mL of Alizarin red S solution in water with concentration of 20 µg/mL was added to each well containing testing specimens. The staining was performed at room temperature for 40 min. After that, the specimens were well washed with plenty of water and examined by optical microscopy (Section 2.6).

## 3. Results and Discussion

### 3.1. Modification of GO with Oligomers or Polymers of Glutamic Acid

Modification of GO was performed via two pathways, namely, “grafting from” the surface and “grafting to” the surface. In the first case, the modification of GO particles with the oligomeric chains of glutamic acid (oligo(Glu)) was achieved via the surface-initiated ring-opening polymerization (ROP) of the γ-benzyl ester of L-glutamic acid N-carboxyanhydride (Glu(OBzl) NCA) with subsequent removal of benzyl protective groups. To introduce the primary amino groups that initiate polymerization, GO was preliminary modified with the amino-bearing linkers, namely L-lysine (Lys) or ethylene diamine (EDA). They were introduced via the reaction of the GO epoxy groups with free amino groups of the linkers. For this purpose, Boc-protected EDA or Lys was used at the first modification step, and then Boc-protective groups were removed to liberate the amine-initiating ROP of NCA. The polymerization was performed by varying the polymerization time (48 and 72 h), temperature (35 and 45 °C), and the excess of the monomer (over epoxy groups of GO).

In the case of the “grafting to” approach, pre-synthesized poly(glutamic acid) (poly(Glu)) was covalently attached to the surface of GO-Lys/GO-EDA particles. For modification of the GO using pre-synthesized poly(Glu), the polymer with DP = 72 and M*_n_* = 11,100 (according to ^1^H NMR spectroscopy) containing 27 mol% of protective groups was used. The immobilization of poly(Glu) was performed using the activated ester method with the activation of 30 or 100% carboxyl groups of the polymer.

**Figure 1:**
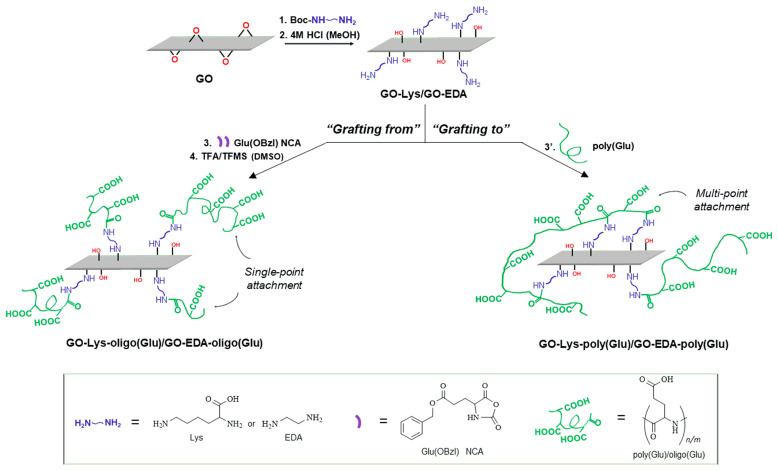
Scheme of GO modification with oligomer/polymer of glutamic acid using “grafting from” or “grafting to” techniques.

The scheme of modifications of GO with the use of “grafting from” and “grafting to” techniques are illustrated in Figure 1. Besides these approaches using different modification chemistry, they also provide different attachments of polymeric chains to the GO surface during modification: while the “grafting from” method allows only a uniform single-point chain attachment, the “grafting to” method provides disordered and multi-point binding to the GO surface.

**Figure 2:**
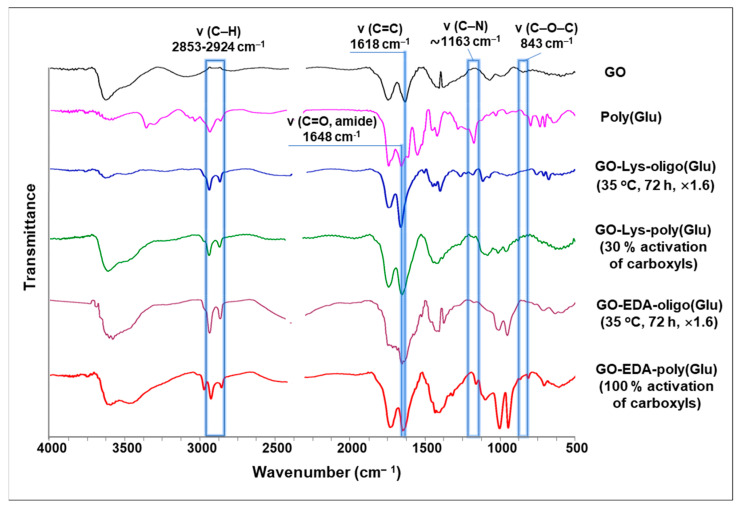
FTIR spectra of GO before and after modification as well as poly(Glu) for comparison.

The modification of GO with oligo/poly(Glu) was testified using Fourier-transform infrared (FTIR) spectroscopy. Figure 2 shows FTIR spectra of the neat and modified GO in comparison with the spectrum of poly(Glu). After grafting, the appearance of characteristic bands in the range of 2853–2924 cm^–1^, attributed to the alkyl fragments of linkers and oligo/poly(Glu), was detected in all samples of modified GO. The appearance of a band at 1163 cm^−1^ for the modified GO is related to the stretching vibrations of C-N in the polypeptide chain. At the same time, a decrease in the intensity or disappearance of a band at 843 cm^−1^ corresponding to C-O-C (epoxy) groups of GO also indicates its modification. The shift of the band maximum at 1618 cm^–1^ to about 1648 cm^–1^, as well as an increase in its intensity, testifies to the oligo/poly(Glu) grafting. These characteristic bands refer to the ν(C=C) GO network and the ν(C=O) amide bond, respectively. The increase in intensity is associated with a partial overlap of these bands. The described signals were observed in all spectra of GO modified with oligo/poly(Glu) that confirm the successful modification using the methods applied.

To evaluate the efficiency of the GO modification with oligo/poly(Glu), the obtained modified GO samples were hydrolyzed at hard acidic conditions to reach the total hydrolysis to free amino acids (Glu and Lys). The contents of free amino acids in hydrolysates were quantitively determined using HPLC analysis. It was found that the acidic removal of Boc-protective groups has a significant effect on the linker content: Lys contents determined in GO-Lys(Boc) and GO-Lys were 15.1 ± 0.9 and 2.1 ± 0.2 µg/mg GO, respectively. In turn, the Lys content in GO-Lys-oligo(Glu) samples obtained after removal of the Bzl group of Glu decreased less significantly compared to GO-Lys deprotection and was 1.6 ± 0.1 µg/mg GO. The data on the Glu content and Glu/Lys ratio depending on the modification conditions are presented in Table 1.

According to the data obtained, polymerization time has an effect on the modification efficiency of grafting from the GO surface. Under other equal conditions, the Glu content increased by 42% when the time was increased from 48 to 72 h. Besides reaction time, the excess of monomer also affected the grafting yield. When the excess of monomer increased from 1.6 to 10 times, the Glu content increased almost twofold. At the same time, the increase in the reaction temperature from 35 and 45 °C had almost no effect on the grafting improvement. In general, the average number of glutamic acid units in the chain under the used conditions varied from 4 to 16.

The use of EDA as a linker resulted in a lower Glu amount in the obtained GO particles. The Glu content determined for the “grafting from” GO-EDA was 5 µg/mg GO, while this for the “grafting from” GO-Lys was 12 µg/mg GO under the same conditions (72 h, 35 °C, and a 1.6-fold excess of monomer). Thus, the use of L-lysine as an amino-containing linker proved to be more effective, which is probably due to the better availability of the amino group on the surface of GO particles due to its better distancing provided by the longer hydrocarbon spacer of Lys (C5) compared to EDA (C2).

In the case of “grafting to” the GO surface with pre-synthesized poly(Glu), the Glu content in the obtained samples was significantly lower compared to the “grafting from” approach. The degree of carboxyl groups activated in poly(Glu), namely 30 or 100%, had no effect on the modification efficacy (Table 1).

Thus, on the one hand, the method of “grafting to” the GO surface, allowing the attachment of the pre-synthesized and characterized polymer, represents a simpler way to modify the GO surface with glutamic acid oligomeric/polymeric chains. On the other hand, a higher Glu content per GO mass unit can be achieved in the case of “grafting from” the surface. The lower Glu content found in the immobilization of poly(Glu) compared to that introduced by the “grafting from” approach can be attributed to the multi-point interaction of an individual macromolecule, which blocks a part of the surface during immobilization and, in turn, hinders the reaction of another polymer chain with surface amino groups.

### 3.2. Characterization of Modified GO-oligo(Glu)/GO-poly(Glu)

The prepared aqueous suspensions of modified GO were characterized in respect to their hydrodynamic characteristics and surface zeta-potential using dynamic and electrophoretic light scattering (DLS and ELS), respectively. The data obtained for various samples are summarized in Table 2. As can be seen, the unmodified GO exhibited a hydrodynamic diameter of around 700 nm and negative zeta-potential close to −43 mV due to the presence of surface carboxylic groups. Modification of GO with Boc-protected Lys as a linker favored the evident aggregation of modified GO in an aqueous medium. In turn, linker deprotection increased the stability of the modified GO in water by providing *D_H_* close to 860–870 nm for the Lys and EDA linkers and by increasing the zeta-potential to −34-(–37) mV through partial compensation of negative charge of surface-linked free amino groups. Grafting and deprotection of glutamic acid oligomers decreased both the hydrodynamic size of the modified GO and zeta-potential that indirectly indicated grafting of the negatively charged polypeptide chains. In the case of the immobilization of the pre-synthesized polymer, larger particles were detected compared to GO modified using the “grafting from” method.

**Figure 3:**
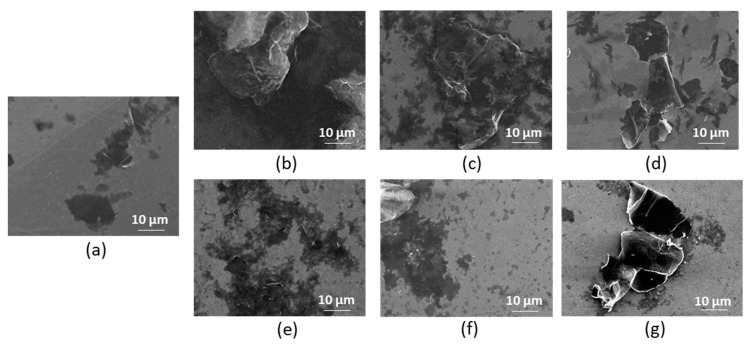
SEM images (×2000) of unmodified and modified GO: neat GO (**a**), GO-EDA (**b**), GO-Lys (**c**), GO-Lys(Boc) (**d**), GO-EDA-oligo(Glu) (sample #3) (**e**), GO-Lys-oligo(Glu) (sample #2) (**f**), and GO-Lys-poly(Glu) (sample #7) (**g**).

The SEM investigation of GO morphology demonstrated the absence of significant changes in particle morphology as a result of modification (Figure 3). Both unmodified and modified samples exhibit a typical GO structure, namely, lamellar with a smooth surface and a low degree of folding of flakes. The modification had no effect on the number of folds. However, the grafting of Lys and EDA amino linkers showed an increase in size due to the aggregation of GO flakes (Figure 3b,c), which can be caused by the interactions between the Lys/EDA amino groups and the oxygen-containing groups of the GO framework. The subsequent modification of GO with oligomeric/polymeric Glu chains shielding the GO backbone and unreacted amino groups, as well as carrying negatively charged carboxylic groups in monomer units, led to a decrease in the flake size due to the resulting electrostatic repulsion (Figure 3e,f). Moreover, the edges of the unmodified GO flake are sharp and easily distinguishable (Figure S2, Supplementary Materials). Conversely, after modification with linker and oligo/poly(Glu), one can see that flakes became less contrasting and boundaries became smoother and more blurred. This is a consequence of the appearance of a sufficiently large amount of aliphatic carbon on the GO surface, which leads to a loss of contrast in the images. This effect becomes more pronounced as the amount of such carbon increases (from GO-linker to GO-linker-oligo/poly(Glu)). In addition, the comparison of the GO flakes modified with the linker before and after deprotection indicates a clear aggregation of the Boc-protected modification (Figure S2, Supplementary Materials). This may be caused by hydrophobic interactions of the Boc-protective groups with each other and with the sp^2^ carbon sites in the graphene that also lead to the folding of the flakes.

In general, the observed differences in size and shape of GO flakes before and after modification are explained by the specific properties of the materials, but not by the method of sample preparation for SEM analysis, which was unified. Thus, the results of the SEM study indirectly confirm the modification of GO and are in agreement with the data obtained from DLS.

In addition, the thermogravimetric analysis (TGA) of the unmodified and modified GO was carried out to monitor possible changes in properties caused by modification (Figure S3 and Table S1, Supplementary Materials). Analysis of the TGA curves revealed the similarity in the thermal degradation profiles of the modified and unmodified GO. For all modified GO samples, significant weight loss was observed in the temperature range of 170–265 °C (31–40% weight loss). For neat GO, a strong weight loss was also observed in this range (of ~33%), but with some narrowing of the region and a shift toward higher temperature values (170–290 °C). A similar weight loss at this step has been also reported for unmodified GO and GO functionalized covalently with D-mannosilated ethylenediamine (man-GO) and related to the oxygen-containing groups of GO [51]. Specifically, the authors reported a weight loss of 38% for GO and 30% for man-GO up to 290 °C.

Slow weight loss of up to 10% was observed in the range of 265–475 °C for all modified GO, while for neat GO the weight loss was less than 3% in the interval of 290–430 °C. The next temperature interval, where a significant weight loss was detected, was 430–570 °C (~45% weight loss) for neat GO and from 440–475 to 650–700 °C (44–50% weight loss) for modified GO samples. For the poly(Glu) used for the grafting, three regions of significant weight loss were established: 190–320 °C (about 38% loss), 330–400 °C (about 18% loss), and 430–560 °C (about 35% loss).

The obtained values of *τ_5_* and *τ_10_*, indicating the 5 and 10% weight loss, were ~182 and ~208 °C for the neat GO (Table S1, Supplementary Materials). At the same time, the *τ_5_* and *τ_10_* values were close to each other for all modified GO samples and varied in the ranges of 161–174 and 182–196 °C, respectively. These results also indirectly confirm the success of GO modification and indicate the possibility of using GO modified with oligo/poly(Glu) as fillers for manufacturing PCL-based composites using a 3D printing technique (PCL melting point is 55–70 °C [52,53]).

### 3.3. Preparation and Characterization of Composite Polymer Films

**Figure 4:**
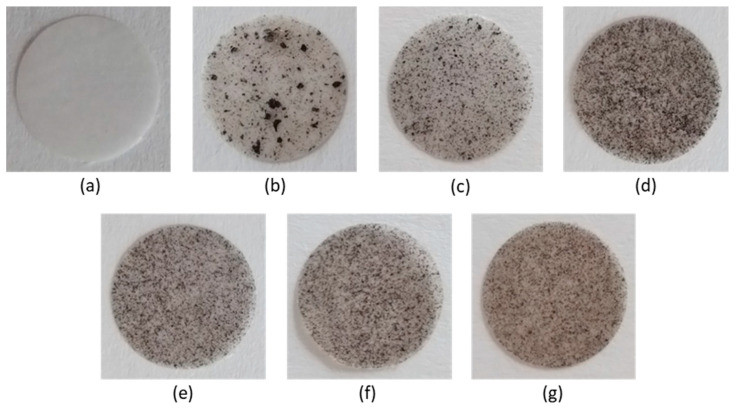
Photos of unfilled PCL (**a**) and PCL-based composite films filled with unmodified and modified GO: PCL/GO (**b**), PCL/GO-Lys-oligo(Glu) #2 (**c**), PCL/GO-Lys-oligo(Glu) #3 (**d**), PCL/GO-Lys-oligo(Glu) #5 (**e**), PCL/GO-Lys-poly(Glu) #6 (**f**), and PCL/GO-EDA-poly(Glu) #9 (**g**). The diameter of the film specimens presented is 10 mm; the content of the filler is 1 wt%.

**Figure 5:**
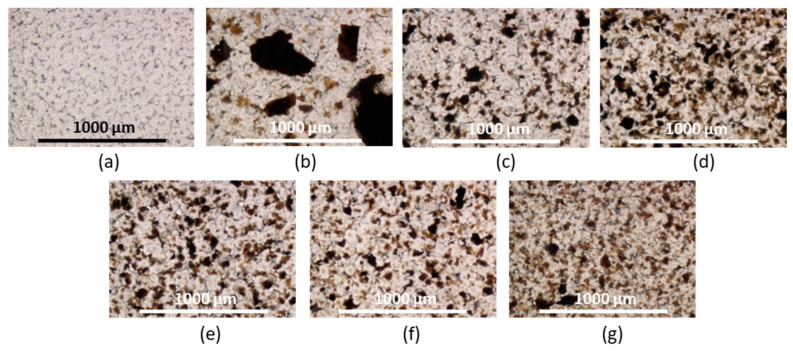
Images of optical microscopy (×4) unfilled PCL (**a**) and PCL-based composite films filled with unmodified and modified GO: PCL/GO (**b**), PCL/GO-Lys-oligo(Glu) #2 (**c**), PCL/GO-Lys-oligo(Glu) #3 (**d**), PCL/GO-Lys-oligo(Glu) #5 (**e**), PCL/GO-Lys-poly(Glu) #6 (**f**), and PCL/GO-EDA-poly(Glu) #9 (**g**). The content of the filler is 1 wt%.

Composite PCL/GO and PCL/modified GO were prepared using a solution casting technique using 0.5 or 1.0 wt% GO/modified GO suspensions in the PCL solution in chloroform. PCL with M*_w_* = 127,000 (*Ð* = 1.81) and intrinsic viscosity (*η*) equal to 1.31 dL/g was applied for the production of composite materials. Some photos of PCL-based composite films filled with unmodified and modified GO are shown in Figure 4. In addition, the optical microscopic images illustrating the surface morphology of the composite specimens are presented in Figure 5. As expected, the modified GO exhibited a more homogeneous distribution in the polymer matrix for all modified samples (Figure 4c–g and Figure 5c–g). At the same time, pronounced aggregation was observed when unmodified GO was used as a filler (Figure 4b and Figure 5b). A similar effect was observed by Valapa et al. for the distribution of unmodified graphene in the PLA matrix [33,54].

The improvement in the distribution of modified GO compared to unmodified one is due to several factors. First, the modification of GO leads to a decrease in the level of hydration of the neat GO, which even after careful drying can absorb moisture. In turn, the quite hydrophilic filler, due to poor compatibility with the hydrophobic matrix of the PCL, tends to aggregate in order to reduce the area of thermodynamically unfavorable contact. As a result of the modification, the hydration and hydrophilicity of GO decrease, and as a consequence, the aggregation intensity of the filler in the hydrophobic environment is reduced. Secondly, unlike hydrophilic GO, which produces stable aqueous suspensions, PGlu shows poor solubility in water without alkalinization, and at the same time in low concentrations or when heated can be dissolved in some organic solvents. Thus, grafted oligo/polymer Glu chains can additionally interact with the aliphatic ester matrix polymer at the supramolecular level, leading to stabilization of the filler in the hydrophobic environment and reduction of its aggregation. As a consequence, more uniform distribution of the modified GO in the matrix can be achieved.

**Figure 6:**
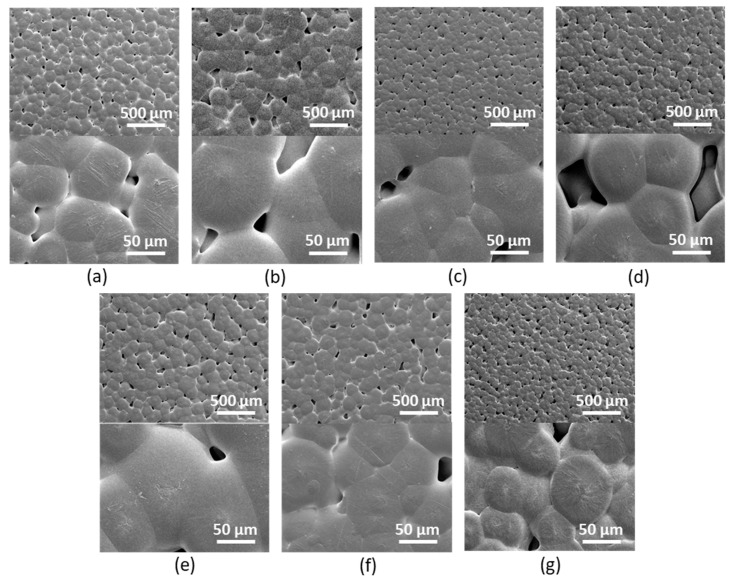
SEM images (top image ×100, bottom image ×1000) of unfilled PCL (**a**) and PCL-based composite films filled with unmodified and modified GO: PCL/GO (**b**), PCL/GO-Lys-oligo(Glu) #2 (**c**), PCL/GO-EDA-oligo(Glu) (**d**), PCL/GO-Lys-oligo(Glu) #5 (**e**), PCL/GO-Lys-poly(Glu) #7 (**f**), and PCL/GO-EDA-poly(Glu) #9 (**g**). The content of the filler is 1 wt%.

The evaluation and comparison of the surface morphology for unfilled PCL and its composites with unmodified and modified GO using SEM revealed a formation of a bit larger polymer clusters and pores for unmodified GO in comparison to unfilled PCL and its composites with modified GO (Figure 6).

**Figure 7:**
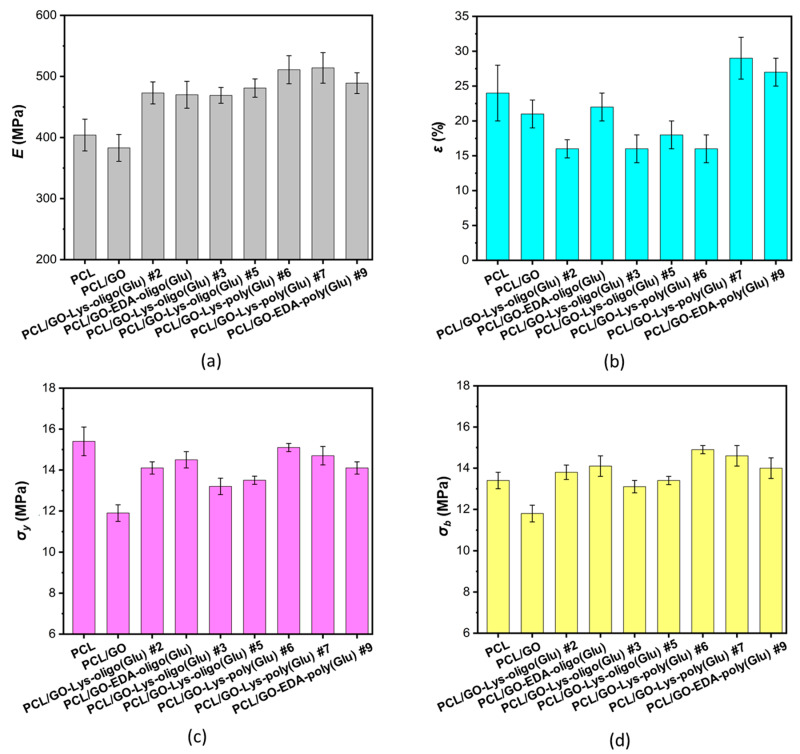
Mechanical characteristics of PCL and its composites with 1 wt% unmodified and modified GO (tensile test): Young’s modulus (*E*) (**a**), elongation at break (*ε_b_*) (**b**), yield strength (*σ_y_*) (**c**), and ultimate tensile strength (*σ_b_*) (**d**).

The mechanical properties of the PCL-based composites filled with unmodified and modified GO were investigated in a tensile test. The determined values of Young’s modulus (*E*), yield strength (*σ_y_*), ultimate tensile strength (*σ_b_*), and elongation at break (*ε_b_*), as well as some deformation curves for composites containing 1 wt% of the filler, are shown in Figure 7 and Figure S4 (Supplementary Materials), respectively. As known, the introduction of a filler into polymer matrices favors an increase in stiffness, namely elastic (Young’s) modulus and tensile yield strength. Most often, simultaneously with this effect, a decrease in elongation at break can occur mainly due to heterogenization of the composite structure and development of the internal local stresses at the interphase “matrix-filler” [55,56,57]. In our case, among the composites containing GO, a noticeable increase in the elastic modulus (of ~18–27% with respect to the unfilled PCL film) was observed for the films containing 1 wt% of modified GO (Figure 7). The change in the content (0.5–1 wt%) of the modified GO as a filler did not have a significant effect on the mechanical characteristics of the obtained composites (Figure 7 and Table S2 of Supplementary Materials). At the same time, the elastic modulus determined for the composites containing unmodified GO was at the level of unfilled PCL material and, in general, the PCL/GO composite showed a tendency to decrease all mechanical characteristics with increasing filler content from 0.5 to 1 wt% (Figure 7 and Table S2 of Supplementary Materials). In some cases, the PCL/modified GO samples still showed an increase in the elastic modulus with increasing filler concentration, while the other parameters did not change.

### 3.4. Cytotoxicity, Osteodifferentiation, and Mineralization Study

#### 3.4.1. In Vitro Cytotoxicity and Proliferation

Biocompatibility of the biomaterials is one of the most important properties for their successful application in medicine. Scaffolds proposed for bone tissue repair should not be cytotoxic to MSCs seeded on their surface before implantation, as well as to cells involved in osteogenesis, such as osteoblasts. Taking this into account, the modified GO was examined for cytotoxicity in human osteosarcoma cells (MG-63 cell line), and the composite materials were studied for the adhesion and proliferation of hMSCs.

**Figure 8:**
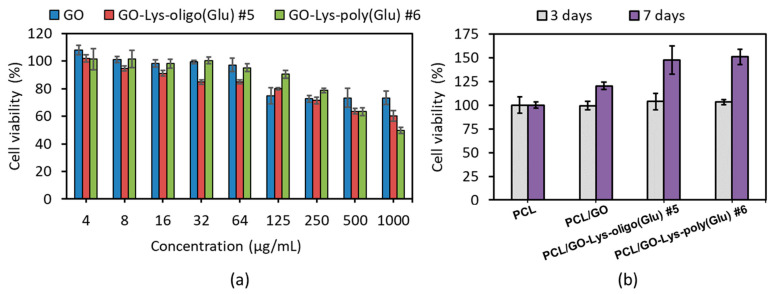
Cytotoxicity (MTT assay) of dispersions of unmodified and modified GO particles in MG-63 cells (3 days) (**a**) and fetal hMSCs proliferation (3 and 7 days) adhered to the surface of PCL-based composite films filled with 1 wt% of modified and unmodified GO (**b**).

MG-63 cells are osteoblast-like and are widely used for similar studies [58,59]. The MTT test was performed using the suspensions of unmodified and modified GO obtained using different approaches. The concentrations of GO were varied from 4 to 1000 μg/mL. GO modified with oligo/poly(Glu) using the approaches “grafting from” and “grafting to”, as well as the neat GO, demonstrate the absence of cytotoxicity against MG-63 cells in the concentration range of 4–250 µg/mL (Figure 8a). At higher concentrations (500–1000 µg/mL), moderate toxicity was observed for modified GO samples. Most likely, this fact can be related to the partial aggregation of the modified GO at high concentration in a multicomponent aqueous system, such as a cell medium, over a long incubation time (72 h). In turn, the precipitation of even a small number of aggregates to the bottom of the well with adhered cells can physically disrupt their normal functioning and contribute to their death.

A comparison of PCL and its composite films filled with 1 wt% of unmodified or modified GO revealed the comparable adhesion of hMSCs (Figure 8b). However, after 7 days of incubation, a slight proliferation was detected for the PCL-based composite filled with GO, whereas the composite filled with grafted with oligo(Glu) showed a more pronounced effect. Similar results on the proliferation of the hMSCs or MG-63 cells adhered to the surface of GO-containing PCL- or PLA-based composites over unfilled PCL were also reported in a number of studies [58,59,60]. Moreover, Scaffaro et al. observed a higher number of cells over time when comparing PCL composites filled with neat GO and covalently PEGylated GO [61].

#### 3.4.2. In Vitro Osteodifferentiation and Mineralization

Before the experiments with cells, to evaluate the ability of the obtained materials to capture calcium ions and form mineral deposits, the mineralization of materials in model media was investigated. For this purpose, the PCL films and composites containing 1 wt% of unmodified or modified GO were sequentially incubated in aqueous solutions of CaCl_2_ (10 mM) and NaH_2_PO_4_ (10 mM) with intermediate washing steps with bidistilled water after each solution. After 9 months of incubation, the films were stained with calcein solution to visualize possible calcium deposits. A significant improvement in calcification was found in the case of the grafted with oligo/poly(Glu) GO-filled composites (Figure S5, Supplementary Materials). An analogous positive effect of poly(Glu) on material mineralization has recently been observed for PLA and PCL composites filled with poly(Glu)-modified nanocrystalline cellulose [47].

In addition to biocompatibility, polymeric composites are considered scaffolds for bone regeneration and should stimulate osteogenic differentiation of hMSCs and accelerate biomineralization. From the results discussed above, it is obvious that the developed composites support the adhesion and growth of hMSCs and can effectively capture calcium ions. To test the osteoconductive properties of the PCL/modified GO composites, osteogenic differentiation of the hMSCs adhered to the surface of the PCL-based films and their composites with unmodified and modified GO was carried out and followed with alkaline phosphatase (ALP) assay after 14 days. The detection of ALP produced by cells, which is known to be one of the early markers of osteodifferentiation [62,63], was performed using BCIP–NBT staining. This analysis is based on hydrolysis of BCIP catalyzed by ALP to produce a blue intermediate. The latter then was oxidized by NBT with the formation of an intense insoluble purple product.

**Figure 9:**
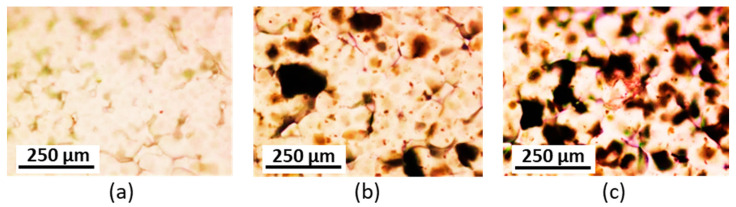
Images of PCL (**a**), PCL/GO (**b**), and PCL/GO-Lys-oligoGlu (#5) (**c**) films after ALP assay after 14 days of the start of osteodifferentiation of fetal hMSCs (optical microscopy, ×10). The intensity of purple color is proportional to the amount of ALP produced by cells.

The images of stained PCL, PCL/GO, and PCL/GO-Lys-oligo(Glu) films are presented in Figure 9. As in the case of mineralization in model media, the less intense staining, reflecting the lowest level of ALP, was observed for pure PCL. The introduction of pure and modified GO into PCL promoted increased ALP production and, therefore, better osteodifferentiation. However, compared to unmodified GO, a higher content of stained zones was observed for the composite containing GO modified by glutamic acid oligomers.

**Figure 10:**
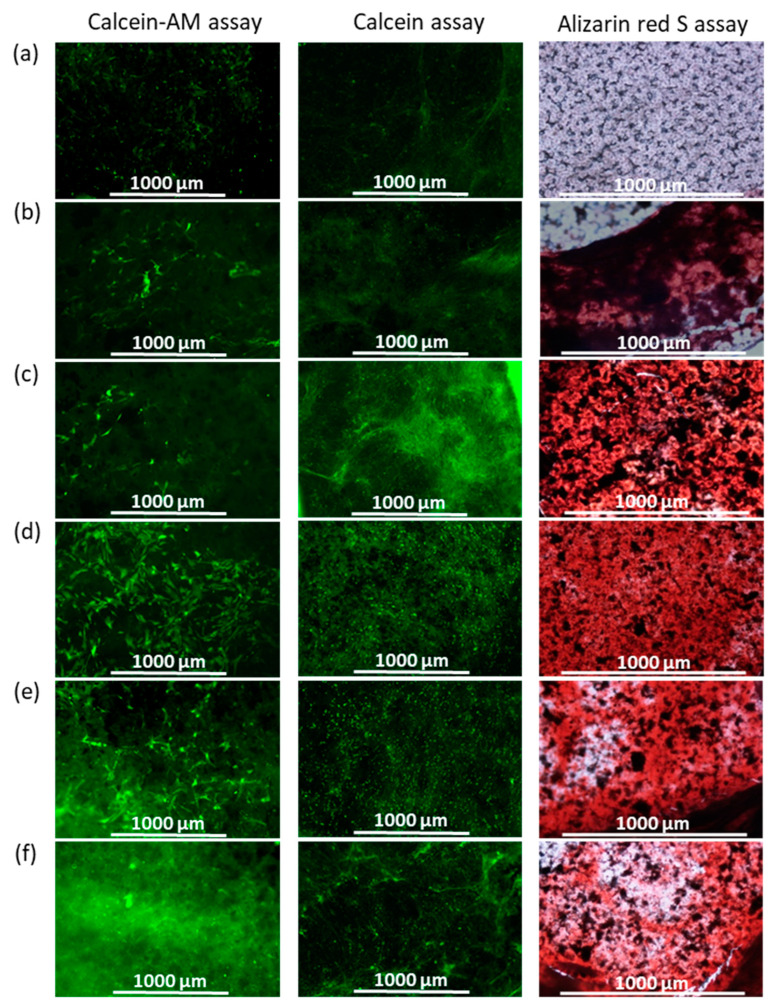
The results of adipose hMSC growth after 10 days of cultivation in expansion medium (calcein–AM assay) and mineral deposit formation after 19 days of osteodifferentiation (calcein and alizarin red S assays): PCL (**a**), PCL/GO (**b**), PCL/GO-Lys-oligo(Glu) #2 (**c**), PCL/GO-Lys-poly(Glu) #7 (**d**), PCL/GO-EDA-oligo(Glu) (**e**), and PCL/GO-EDA-poly(Glu) #9 (**f**). The intensity of green (for calcein–AM and calcein) and red (for alizarin red S) color corresponds to the content of living cells (for calcein–AM) and mineral deposits (for calcein and alizarin red S) on the surface of materials (fluorescence and optical microscopy, ×4). The content of the filler is 1 wt%.

For comparison of the ability of different materials to biomineralization, PCL films and their composites with unmodified and functionalized GO bearing the adhered hMSCs were cultured for 10 days in an expansion medium and then transferred to the osteogenic differentiation medium (ODM). After 19 days of cultivation in ODM, a number of films were stained with calcein, while another one was stained with alizarin red S to visualize the calcium deposits. In addition, the efficiency of cell proliferation on the surface of different materials under study was visualized using calcein–AM staining of cells before the medium was replaced with ODM. Images obtained by fluorescent (calcein–AM or calcein staining) or optical (alizarin red S staining) microscopy are presented in Figure 10.

As can be seen, the control unfilled PCL films showed a low level of proliferation and almost no biomineralization. The PCL/GO composites also maintained a low level of cell growth, but at the same time, osteodifferentiation and biomineralization effects were observed compared to pure PCL. However, in the most intensive cell growth, osteodifferentiation and biomineralization were detected for all composites containing oligo/poly(Glu)-functionalized GO. This result is consistent with the results discussed above regarding cell proliferation and mineralization in the model media and ALP assay.

Furthermore, a similar effect on improved biomineralization for the oligo/poly(Glu)-containing materials was reported in several papers [43,64,65]. In particular, Karaman et al. indicated a positive effect of glutamic acid peptides bound to PLA/PLGA nanofibers on calcium phosphate nucleation and osteogenic differentiation of bone marrow stromal cells [64]. Averianov et al. reported improved in vitro [65] and in vivo biomineralization [43] when nanocrystalline cellulose modified with poly(Glu) was used as a filler to PLA or PCL.

In all discussed cases, the enhanced deposition of calcium salts is attributed to the improved capture of calcium ions by the carboxylic groups of oligo/poly(Glu) introduced into the aliphatic polyester materials directly or as a part of the filler.

## 4. Conclusions

In this study, two different pathways for covalent GO modification with oligomers or polymers of glutamic acid were proposed. Specifically, the grafting of oligo(Glu) chains from the surface of amino-modified GO was performed via ring-opening polymerization of N-craboxyanhydride of γ-benzyl ester of glutamic acid. This technique guarantees the single-point attachment of the oligomer chains on the GO surface. Another approach was based on grafting the pre-synthesized polymer to the surface of amino-modified GO via the activated ester method. In contrast to the first approach, the multi-point attachment of the polymer to the GO surface is random. The resulting modifications were tested using FTIR spectroscopy and qualitative amino acid HPLC analysis. It was found that increasing the polymerization time, temperature, and monomer excess promotes the grafting efficacy in the case of the “grafting from” approach. At the same time, changing the degree of activation of the poly(Glu) carboxylic groups had no effect on the GO modification process. The average hydrodynamic diameter of GO particles slightly decreased after modification with oligo/poly(Glu). In addition, TGA and SEM results also indirectly indicated in favor of GO modification. PCL-based composites filled with modified GO revealed slight improvements in tensile properties, whereas no improvement was observed when neat GO was used as a filler. In vitro biological experiments confirmed the low cytotoxicity of GO and its derivatives, as well as the enhanced proliferation of hMSCs on the surface of composites containing modified GO. Moreover, composites containing GO modified with oligo/poly(Glu) demonstrated evident osteoconductive properties compared to PCL/GO composites.

Taking into account the stability of modified GO at the melting temperature of PCL, it can be used as a filler for the production of 3D-printed PCL-based composite scaffolds using the extrusion method of additive manufacturing. GO functionalization using Lys as a linker appears to be the optimal modification route for both approaches of grafting oligo/polyGlu. Samples of the modified GO #5 (“grafting from”) and #6/7 (“grafting to”) can be considered optimal for further use as fillers for the manufacturing of composites via 3D printing. The 3D-printed scaffolds based on the developed osteoconductive composites have great potential for further evaluation of their bone regeneration potential in vivo.

## Figures and Tables

**Table 1:** Content of Glu and Glu/Lys molar ratio determined for GO-Lys-oligo(Glu)/GO-Lys-poly(Glu) modification at different conditions (quantitative amino acid HPLC analysis of hydrolyzates).

Sample		Conditions				Glu Content (µg/mg GO) ^b^	Glu/Lys Ratio (mol/mol)
		Temperture (°C)	Reaction Time (h)	Monomer Excess ^a^	Activation of Poly(Glu) (%)		
GO-Lys-oligo(Glu) (“grafting from”)	#1	35	48	1.6	−	7	4
	#2	35	72	1.6	−	12	7
	#3	45	72	1.6	−	13	8
	#4	45	72	4.7	−	19	12
	#5	45	72	10	−	25	16
GO-Lys-poly(Glu) (“grafting to”)	#6	22	3	−	30	6	3
	#7	22	3	−	100	6	3

^a^ Monomer excess relative to epoxy groups of GO; ^b^ Relative standard deviation in the range was 7–9%.

**Table 2:** Hydrodynamic diameters and indices of polydispersity (DLS, 25 °C, dispersion in alkalized water (pH 7.5), 0.5 mg/mL) as well as zeta-potential (ELS, 25 °C, dispersion in water, 0.05 mg/mL) of GO and its derivative ELS.

Sample		Grafting Conditions	D_H_ (nm)	PDI	Zeta-Potential (mV)
GO		−	725 ± 33	0.28	−42.9 ± 0.5
GO-Lys(Boc)		−	1547 ± 195	0.40	−42.4 ± 2.3
GO-Lys		−	861 ± 34	0.43	−34.1 ± 2.4
GO-EDA		−	869 ± 63	0.36	−37.6 ± 8.0
GO-Lys-oligo(Glu(OBzl))		48 h, 35 °C, ×1.6 *	1244 ± 61	0.57	−34.8 ± 5.1
GO-Lys-oligo(Glu)	#1	48 h, 35 °C, ×1.6 *	518 ± 26	0.70	−46.1 ± 1.4
GO-Lys-oligo(Glu)	#4	72 h, 45 °C, ×4.7 *	372 ± 47	0.65	−42.7 ± 0.8
GO-Lys-oligo(Glu)	#5	72 h, 45 °C, ×10 *	465 ± 18	0.69	−42.1 ± 1.7
GO-Lys-poly(Glu)	#6	30% activation of poly(Glu) carboxyls	672 ± 26	0.56	−41.1 ± 4.1
GO-Lys-poly(Glu)	#7	100% activation of poly(Glu) carboxyls	898 ± 36	0.38	−40.5 ± 2.3
GO-EDA-oligo(Glu)	#8	48 h, 35 °C, ×1.6 *	589 ± 29	0.43	−38.8 ± 2.7
GO-EDA-poly(Glu)	#9	100% activation of poly(Glu) carboxyls	922 ± 43	0.33	−40.6 ± 0.7

* Monomer excess relative to epoxy groups of GO.

## Data Availability

The data are available within the article and its Supplementary Materials.

## Supplementary Materials

The following supporting information can be downloaded at: https://www.mdpi.com/article/10.3390/polym15122714/s1; Figure S1: Scheme of the composite film manufacturing; Figure S2: SEM images (×2000) of unmodified and modified GO: (a) GO, (b) GO-EDA, (c) GO-Lys, (d) GO-Lys(Boc), (e) GO-EDA-oligo(Glu) (48 h, 35 °C, 1.6-fold excess of monomer), (f) GO-Lys-oligo(Glu) (72 h, 35 °C, 1.6-fold excess of monomer), (g) GO-Lys-oligo(Glu(OBzl)) (72 h, 35 °C, 1.6-fold excess of monomer); Figure S3: TGA curves of unmodified and modified GO as well as poly(Glu); Figure S4: Deformation curves (tensile test) for neat and some composite PCL films containing unmodified/modified GO particles; Figure S5: Mineralization study of the neat PCL (a) and its composites with unmodified (b) and modified GO (PCL/OG-Lys-oligo(Glu) #2 (c), PCL/GO-Lys-poly(Glu) #7 (d), PCL/GO-EDA-oligo(Glu) (e), PCL/GO-EDA-poly(Glu) #9 (f)) in the model media after 9 months of exposure; Table S1: Temperature values at 5 (τ_5_) and 10% (τ_10_) weight loss of modified GO compared to unmodified GO and poly(Glu); Table S2: Mechanical properties PCL-based composites with 0.5 wt% unmodified and modified GO (tensile test).
