# Supplementary material for: Composites Based on Poly(ε-caprolactone) and Graphene Oxide Modified with Oligo/Poly(Glutamic Acid) as Biomaterials with Osteoconductive Properties
Source: Polymers (Basel). 2023 Jun 17;15(12):2714. doi: 10.3390/polym15122714 (PMC10301562; doi:10.3390/polym15122714)
Supplement: Supplementary file 1 [file polymers-15-02714-s001.zip › polymers-2425265-supplementary.pdf]

polymers

Supplementary Materials
Composites Based on Poly(ε-caprolactone) and Graphene Oxide Modified with Oligo/Poly(Glutamic Acid) as Biomaterials with Osteoconductive Properties

Olga Solomakha [1], Mariia Stepanova [1], Iosif Gofman [1], Yuliya Nashchekina [2], Maxim Rabchinskii [3], Alexey Nashchekin [3], Antonina Lavrentieva [4] and Evgenia Korzhikova-Vlakh [1,*]

[1] Institute of Macromolecular Compounds, Russian Academy of Sciences, St. Petersburg 199004, Russia; solomanya@bk.ru (O.S.); maristepanova@hq.macro.ru (M.S.); gofman@imc.macro.ru (I.G.);
[2] Institute of Cytology, Russian Academy of Sciences, St. Petersburg 194064, Russia; ulychka@mail.ru
[3] Ioffe Institute, Politekhnicheskaya st. 26, St. Petersburg 194021, Russia; rabchinskii@mail.ioffe.ru (M.R.); nashchekin@mail.ioffe.ru (A.N.)
[4] Institute of Technical Chemistry, Leibniz University of Hannover, 30167 Hannover, Germany; lavrentieva@iftc.uni-hannover.de
* Correspondence: vlakh@hq.macro.ru

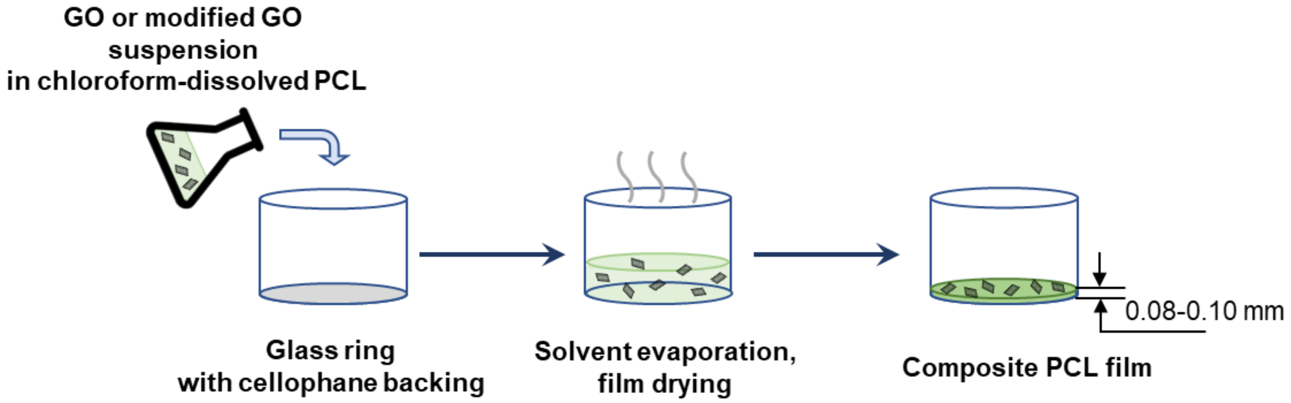

Figure S1. Scheme of the composite film manufacturing.

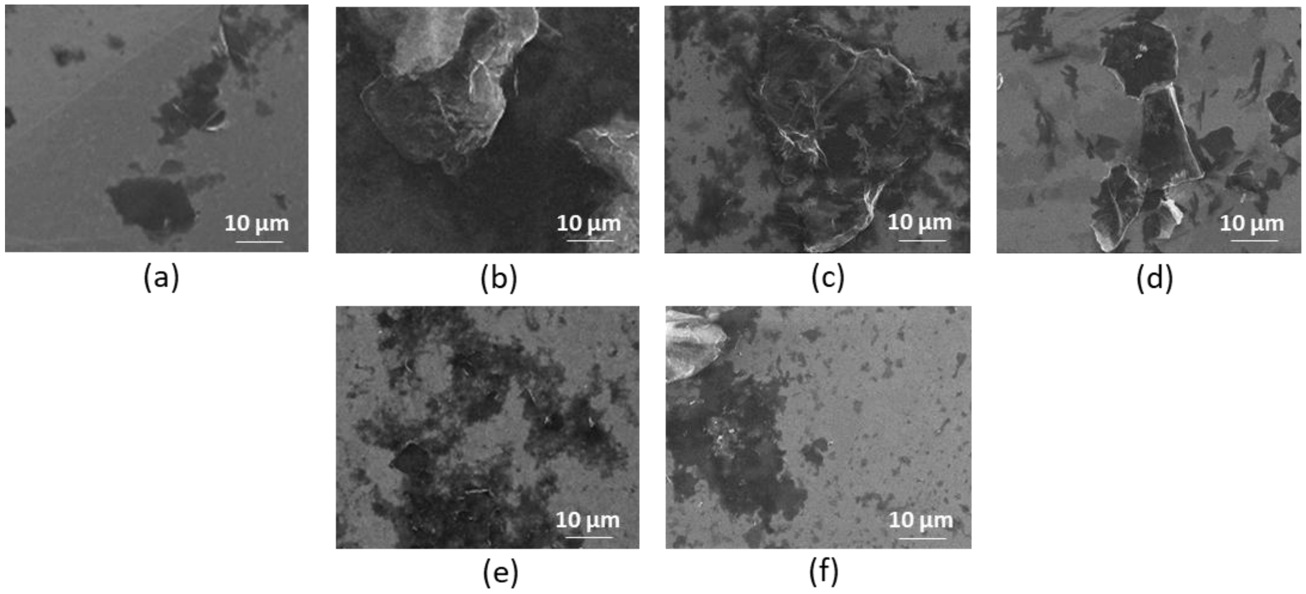

Figure S2. SEM images (×2000) of unmodified and modified GO: (**a**) GO, (**b**) GO-EDA, (**c**) GO-Lys, (**d**) GO-Lys(Boc), (**e**) GO-EDA-oligo(Glu) (48 h, 35 °C, 1.6-fold excess of monomer), (f) GO-Lys-oligo(Glu) (72 h, 35 °C, 1.6-fold excess of monomer), (g) GO-Lys-oligo(Glu(OBzl)) (72 h, 35 °C, 1.6-fold excess of monomer).

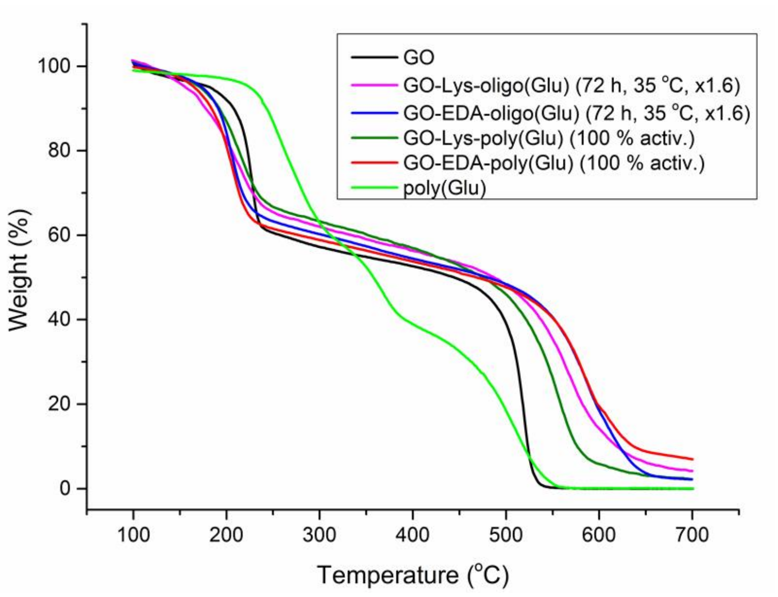

Figure S3. TGA curves of unmodified and modified GO as well as poly(Glu).

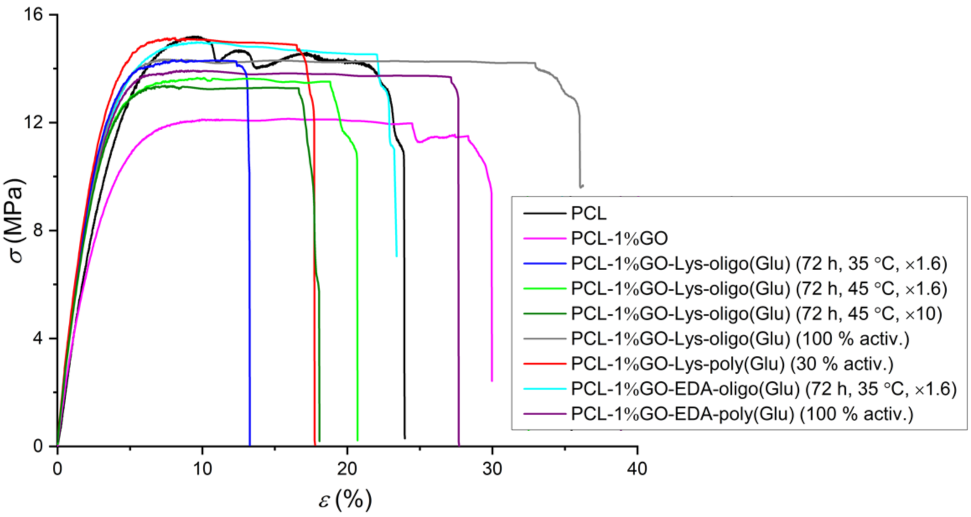

Figure S4. Deformation curves (tensile test) for neat and some composite PCL films containing unmodified/modified GO particles.

Table S1. Temperature values at 5 *(τ_5)* and 10 % *(τ_{10})* weight loss of modified GO compared to unmodified GO and poly(Glu).

Sample	Varying grafting conditions	τ_5 (°C)*	τ_{10} (°C)*
GO	-	182	208
GO-Lys	-	186	212
GO-EDA	-	188	208
GO-Lys-oligo(Glu)	48 h, 35 °C, ×1.6	163	192
GO-Lys-oligo(Glu)	72 h, 35 °C, ×1.6	153	177
GO-Lys-oligo(Glu)	72 h, 45 °C, ×1.6	162	182

GO-Lys-oligo(Glu)	72 h, 45 °C, ×4.7	161	187
GO-Lys-oligo(Glu)	72 h, 45 °C, ×10	164	189
GO-EDA-oligo(Glu)	48 h, 35 °C, ×1.6	163	183
GO-EDA-oligo(Glu)	72 h, 35 °C, ×1.6	174	191
GO-Lys-poly(Glu)	30 % activation of poly(Glu) carboxyls	168	196
GO-Lys-poly(Glu)	100 % activation of poly(Glu) carboxyls	172	192
GO-EDA-poly(Glu)	100 % activation of poly(Glu) carboxyls	165	184
poly(Glu)	-	236	248

*Relative standard deviation was 1-2 %.

Table S2. Mechanical properties PCL-based composites with 0.5 wt% unmodified and modified GO (tensile test).

Sample	E (MPa)	σ_y (MPa)	σ_b (MPa)	ε_b (%)
PCL-0.5%GO	405 ± 12	12.5 ± 0.2	12.2 ± 0.2	24 ± 2
PCL-0.5%GO-Lys-oligo(Glu) (72 h, 35 °C, ×1.6)	447 ± 12	13.7 ± 0.3	13.5 ± 0.5	22 ± 3
PCL-0.5%GO-EDA-oligo(Glu) (72 h, 35 °C, ×1.6)	422 ± 17	14.7 ± 0.4	14.3 ± 0.4	29 ± 2
PCL-0.5%GO-Lys-poly(Glu) (100 % activation of poly(Glu) carboxyls)	448 ± 19	14.1 ± 0.4	14.0 ± 0.4	28 ± 2
PCL-0.5%GO-EDA-poly(Glu) (100 % activation of poly(Glu) carboxyls)	455 ± 16	13.8 ± 0.5	13.4 ± 0.5	28 ± 3

Mineralization Study in Model Media

The mineralization study was performed by sequential incubation of the composite film specimens in 10 mM solutions of $CaCl_2$ and NaH_2PO_4. For this purpose, the films of 6 mm in diameter were glued onto the bottom of a 24-well plate using BF-6 glue. The wells containing specimens were filled with a 1 mL $CaCl_2$ solution and the plate was incubated within 2 days at 37 °C. After that, fixed specimens were carefully washed with the excess of bidistilled water, and then incubated in 1 mL NaH_2PO_4 for another 2 days at 37 °C. This cycle was repeated for 9 months. Finally, washed and air-dried films were stained with calcein solution to visualize the mineral deposits.

Staining with calcein was carried out exactly as described in section 2.8.3, except that the dye solutions were applied directly to each unglued film placed in a glass vial. The volume of dye solutions was 0.5 mL. After removing the dye and washing, the films were examined using fluorescence microscopy (section 2.6). Calcein fluorescence was detected at the emission wavelength 530 nm (an excitation wavelength is 480 nm).

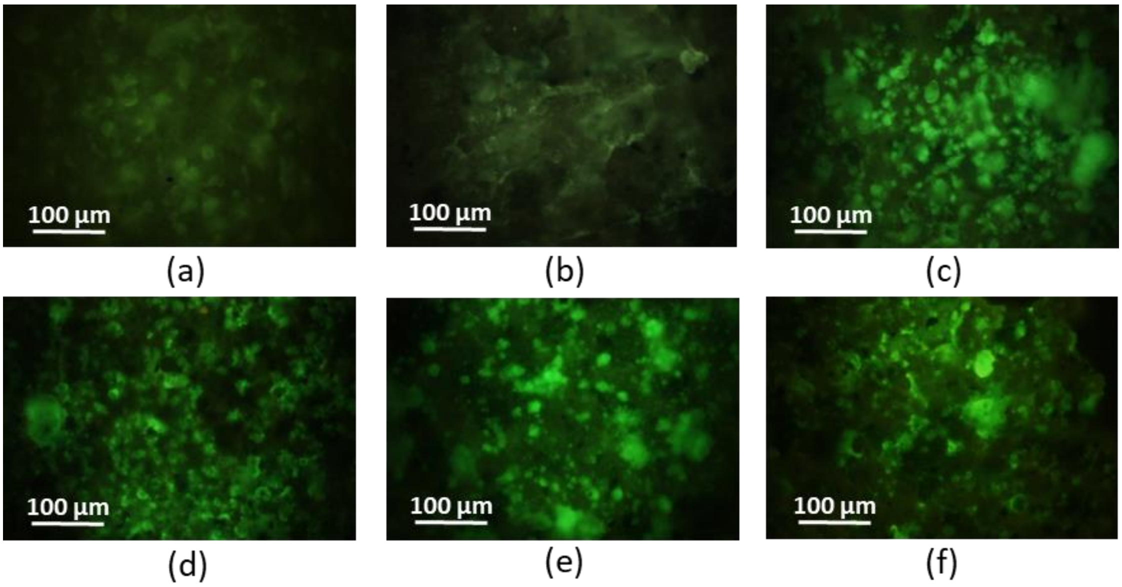

Figure S5. Mineralization study of the neat PCL **(a)** and its composites with unmodified **(b)** and modified GO (PCL/OG-Lys-oligo(Glu) #2 (**c**), PCL/GO-Lys-poly(Glu) #7 (**d**), PCL/GO-EDA-oligo(Glu) (**e**), PCL/GO-EDA-poly(Glu) #9 (**f**)) in the model media after 9 months of exposure. The intensity of green color (staining with calcein) indicates the content of calcium deposits on the surface of the materials (fluorescence microscopy, ×20). The content of the filler is 1 wt%.
